# Genotype–Phenotype Links Between Aminoglycoside-Modifying Enzymes and Aminoglycoside MICs in Aminoglycoside-Resistant *Klebsiella pneumoniae* in a Southern Vietnam Tertiary Hospital

**DOI:** 10.3390/microorganisms14020463

**Published:** 2026-02-13

**Authors:** Tuan Huu Ngoc Nguyen, Huy Quang Nguyen, Tham Thi Hong Ho, Hung Cao Dinh, Huong Thi Nguyen, Tam Bui Thanh Pham, Ha Minh Nguyen

**Affiliations:** 1 Biomedical Research and Diagnostics Center, Pham Ngoc Thach University of Medicine, Ho Chi Minh City 700000, Vietnam; nhntuan@pnt.edu.vn (T.H.N.N.); huynq@pnt.edu.vn (H.Q.N.); thamhth@pnt.edu.vn (T.T.H.H.); 2 Medical Biochemistry & Molecular Biology Department, Fundamental Sciences and Basic Medical Sciences, Pham Ngoc Thach University of Medicine, Ho Chi Minh City 700000, Vietnam; 3 Department of Internal Medicine, Pham Ngoc Thach University of Medicine, Ho Chi Minh City 700000, Vietnam; hungcd@pnt.edu.vn; 4 Laboratory Department, Nguyen Tri Phuong Hospital, Ho Chi Minh City 700000, Vietnam

**Keywords:** antibiotic resistance, Vietnam, multidrug-resistant strains, hospital-acquired infections, community-acquired infections, minimum inhibitory concentrations

## Abstract

Aminoglycosides remain important components of combination therapy for complicated *Klebsiella pneumoniae* infections in Vietnam; however, gene-level evidence linking aminoglycoside-modifying enzymes (AMEs) with minimum inhibitory concentrations (MICs) and multidrug resistance is limited, particularly in tertiary-care settings in southern Vietnam. A cross-sectional analysis was conducted on 186 non-duplicate aminoglycoside-resistant *Klebsiella pneumoniae* clinical isolates collected in a tertiary-care hospital in Ho Chi Minh City. Species identity was reconfirmed using ZKIR qPCR. MICs for amikacin, gentamicin, and tobramycin were determined by broth microdilution according to Clinical and Laboratory Standards Institute (CLSI) guidelines, and 14 AME genes were detected using targeted qPCR. Associations between AME genes, aminoglycoside MICs or susceptibility categories, and co-resistance to major antibiotic classes were assessed using non-parametric and exact tests with Benjamini–Hochberg false discovery rate correction, with emphasis on effect direction and clinically interpretable genotype–phenotype patterns beyond statistical significance alone. AME genes were highly prevalent, with *ant(2″)-Ia*, *aac(6′)-Ir*, and *aac(6′)-Ib* detected in 97.3%, 91.9%, and 89.8% of isolates, respectively. The presence of *aac(6′)-Ib* and *aac(6′)-Ih_v* was associated with higher aminoglycoside MICs, resistance to amikacin and tobramycin, and broader multidrug resistance, including carbapenem resistance, whereas several other AMEs were linked to lower MICs. These findings indicate that specific AME profiles, particularly *aac(6′)-Ib* and *aac(6′)-Ih_v*, are associated with intensified aminoglycoside resistance in this setting and support the need for gene-informed surveillance to prioritise confirmatory MIC-based Antimicrobial Susceptibility Testing (AST) to guide local antimicrobial stewardship.

## 1. Introduction

Carbapenem-resistant *Klebsiella pneumoniae* is a WHO priority pathogen and a major driver of healthcare-associated infections worldwide, resulting in substantial morbidity, mortality, and costs in both high- and low-resource settings [1,2]. Therapeutic options are increasingly constrained by the production of Extended-Spectrum Beta-Lactamase (ESBLs) and carbapenem resistance; amid limited access to newer agents in many settings, clinicians continue to rely on aminoglycosides (e.g., gentamicin, tobramycin, amikacin) as part of empirical and targeted regimens [3,4]. In Vietnamese tertiary-care hospitals, this reliance persists despite high multidrug resistance and constrained access to newer antimicrobial agents.

Aminoglycoside resistance in *K. pneumoniae* is dominated by aminoglycoside-modifying enzymes (AMEs)—acetyltransferases (*aac*), nucleotidyltransferases (*ant*), and phosphotransferases (*aph*)—which inactivate drugs and are frequently carried on mobile genetic elements that facilitate rapid spread [5,6]. International studies show considerable heterogeneity in AME gene prevalence and composition across regions and healthcare contexts, with commonly detected genes including *aac(6′)-Ib*, *aac(3′)-II*, *ant(2″)-Ia*, and *aph(3′)-Ia*; reported rates vary widely (≈20–80% or higher) depending on local epidemiology and antibiotic selection pressures [7,8,9]. These gene-level data are clinically relevant: AME profiles can correlate with higher minimum inhibitory concentrations (MICs) and multidrug-resistant phenotypes, thereby informing empirical therapy, escalation/de-escalation decisions, and stewardship priorities [9,10,11]. Accordingly, emphasis on effect direction and genotype–phenotype interpretability (e.g., which AME signatures track with elevated MIC distributions and broader co-resistance) is important beyond statistical significance alone.

Vietnamese evidence has largely emphasized phenotypic resistance trends in *K. pneumoniae* across hospitals, documenting high aminoglycoside nonsusceptibility alongside ESBL and carbapenem resistance [12]. However, systematic molecular characterization of AME genes in *K. pneumoniae* in Vietnam remains scarce. In particular, the distribution and co-occurrence of AME genes among aminoglycoside-resistant isolates, as well as whether gene distributions and MICs differ between community-acquired infections (CAI) and hospital-acquired infections (HAI), remain poorly defined in southern Vietnam. This constitutes a gap for the research community rather than an individual agenda: current national data are insufficient to guide gene-informed surveillance, empirical therapy, and infection control strategies in Vietnamese hospitals.

Addressing this gap is operationally crucial in resource-limited settings. Gene-level surveillance can (i) refine empirical choices when rapid diagnostics are unavailable, (ii) identify AME configurations linked to high-level or broad aminoglycoside resistance that may undermine common combinations, and (iii) support targeted infection-control responses where mobile resistance determinants are propagating within wards or along referral networks. At the same time, AME detection should be interpreted as adjunctive to phenotypic MIC testing, because genotype–phenotype relationships may vary by allele/substrate and co-occurring resistance mechanisms [13,14]. Generating local molecular data is therefore integral to antimicrobial stewardship and to preserving aminoglycoside utility where alternatives are limited.

This study aims to determine the prevalence and distribution of AME genes among aminoglycoside-resistant *K. pneumoniae* isolates in a southern Vietnamese tertiary hospital and to compare AME gene distributions and aminoglycoside MICs between community- and hospital-acquired infections. In addition, we assess whether specific AME profiles, particularly key determinants commonly implicated in elevated MICs, are associated with intensified aminoglycoside resistance and broader co-resistance backgrounds in this setting.

## 2. Material and Methods

### 2.1. Study Design and Setting

We conducted a cross-sectional laboratory study of aminoglycoside-resistant *Klebsiella pneumoniae* recovered from routine clinical specimens at a tertiary public hospital in Ho Chi Minh City, Viet Nam, during calendar year 2023. Isolates were processed and tested at the Biomedical Research Center, Pham Ngoc Thach University of Medicine.

### 2.2. Sample Size and Sampling Strategy

The minimum sample size for estimating a single proportion was calculated using $n=\frac{Z^{2}\times p\left(1-p\right)}{d^{2}}$, with Z = 1.96 for 95% confidence, a conservative prevalence estimate of *p* = 0.50 (which maximizes variance given heterogeneous AME gene prevalences in the literature), and a margin of error d = 0.08. This yields N = 150. Allowing ~10% non-evaluable isolates (e.g., non-viable, identification failure), the target minimum became N = 167. The study ultimately analyzed 186 eligible resistant isolates for AME genes, thus exceeding the calculated minimum. Isolate accrual used consecutive convenience sampling of all non-duplicate *K. pneumoniae* meeting eligibility criteria during the study period.

### 2.3. Inclusion and Exclusion Criteria

Inclusion criteria were as follows: (i) clinical isolates of *K. pneumoniae* obtained in 2023; (ii) non-duplicate isolates, defined as selecting a single isolate per patient—if multiple *K. pneumoniae* isolates were identified from the same individual, only the earliest eligible isolate within the episode was included; and (iii) demonstration of phenotypic resistance to gentamicin and/or tobramycin based on initial hospital laboratory screening.

Exclusion criteria included: (i) duplicate patient isolates; (ii) isolates with ambiguous MICs, such as those yielding uninterpretable colorimetric results or exhibiting suspected contamination that persisted following re-testing; and (iii) isolates confirmed to be susceptible to all three target aminoglycosides (amikacin, gentamicin, tobramycin) upon broth microdilution confirmation.

### 2.4. Isolate Identification and Confirmation

Species identification at the hospital laboratory was performed using standard biochemical methods. Genomic DNA was then extracted, and species identity was verified by ZKIR real-time PCR [15]; isolates that failed confirmation were excluded. Complete primer sequences and cycling conditions are provided in Supplementary Table S1. Manufacturers and catalogue numbers for key reagents/consumables and instruments are provided in Supplementary Table S2.

### 2.5. Antimicrobial Susceptibility Testing (and MIC Determination)

MICs for amikacin, gentamicin, and tobramycin were determined by broth microdilution in cation-adjusted Mueller–Hinton broth following Clinical and Laboratory Standards Institute (CLSI) M07 procedures; interpretive categories used CLSI M100 (2025) breakpoints [16,17]. Briefly, two-fold serial dilutions were prepared to cover breakpoint-relevant concentration ranges. Plates were inoculated according to CLSI inoculum specifications, incubated at 35 ± 2 °C for the recommended duration, and MICs were read as the lowest concentration preventing visible growth. *E. coli* ATCC 25922 served as the quality-control strain within each testing run. All MIC tests were performed in technical duplicate; discordant results triggered a repeat run. Manufacturers and catalogue numbers for antimicrobial standards, media, and consumables are provided in Supplementary Table S3.

### 2.6. Detection of Aminoglycoside-Modifying Enzyme (AME) Genes

AME genes from the *aac*, *ant*, and *aph* families were interrogated by SYBR-based qPCR using the QuantStudio System (Thermo Fisher Scientific, Waltham, MA, USA) [18]. No-template controls were included for every target in each run; positive detection required a single specific melt peak of the expected product. All qPCR reactions were run in duplicate, with repeats to account for potential discordance. Amplifications with non-specific melt profiles or inconsistent replicate results were repeated, and only concordant results were retained for analysis. Primer sequences, reaction volumes, and thermal profiles are listed in Supplementary Table S1.

### 2.7. Variables and Data Handling

The recorded variables comprised infection source CAI or HAI, specimen type, antimicrobial susceptibility testing (AST)-derived resistance phenotypes (aminoglycosides, ESBL, imipenem), minimum inhibitory concentration (MIC) values (µg/mL), presence or absence of aminoglycoside-modifying enzyme (AME) genes, and the total gene count per isolate.

Infection sources were classified as follows:

• CAI: Defined as an infection diagnosed within 48 h of hospital admission in patients without prior healthcare exposure during the look-back period specified in the standard operating procedures (SOPs), based on clinician diagnosis and chart review.
• HAI: Defined as an infection diagnosed 48 h or more after admission, or associated with recent healthcare interventions, as determined by electronic medical record review and the treating team’s diagnosis.

**Figure 1:**
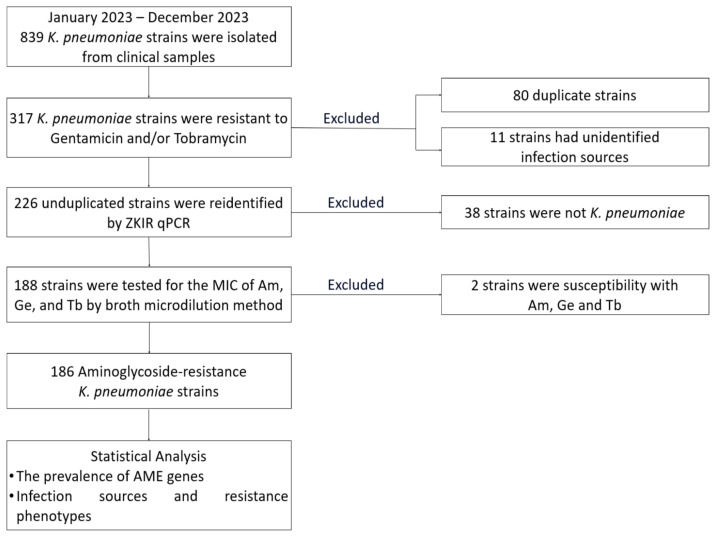
Flowchart of sample collection and pathogen identity verification.

MIC readings of “>64” µg/mL were coded as 128 µg/mL for distributional summaries. This recoding was applied for descriptive distributional summaries and non-parametric comparisons, and does not affect categorical CLSI interpretations. The data management steps and isolated flow are illustrated in Figure 1.

### 2.8. Statistical Analysis

Analyses were performed using Stata 14.2. Descriptive proportions were presented as percentages with 95% CI (Wilson). The normality of MIC distributions was evaluated using the Shapiro–Wilk test; because MICs were non-normal, nonparametric tests were applied according to prespecified criteria. Prevalence comparisons of AME genes among groups utilised the Chi-square test (where expected counts were ≥5) or the two-sided Fisher’s exact test (for counts < 5). MIC comparisons based on gene presence/absence or clinical categories employed the Mann–Whitney U test for two-group analyses and the Kruskal–Wallis test for analyses involving three or more groups. Multiple comparisons were addressed using the Benjamini–Hochberg false discovery rate (FDR) procedure, with statistical significance defined as an FDR-adjusted *p*-value (q-value) less than 0.05. Effect estimates (including proportions and medians with interquartile ranges) are presented alongside their respective *p*-values; no data imputation was necessary. To improve clinical interpretability, predictive performance metrics (sensitivity, specificity, positive predictive value, negative predictive value, and likelihood ratios) were additionally calculated for selected high-priority AME signatures (including *aac(6′)-Ib* and *aac(6′)-Ih_v*) for aminoglycoside resistance and/or high MIC outcomes.

### 2.9. Ethics

This study was approved by the Ethical Committee of Nguyen Tri Phuong Hospital, Ho Chi Minh City, Viet Nam (Decision Number 2640/NTP-HĐĐĐ; 30 November 2023). Use of anonymized clinical isolates obtained during routine care led the committee to waive informed consent. All procedures were conducted in accordance with the Declaration of Helsinki.

## 3. Results

### 3.1. Sample Description

A total of 186 non-duplicate aminoglycoside-resistant *Klebsiella pneumoniae* isolates were analyzed (Table 1). Most isolates were from HAI (116/186, 62.4%, 95% CI [55.2–69.0%]), with CAI accounting for 70/186 (37.6%, 95% CI [31.0–44.8%]). The lower respiratory tract was the predominant specimen source (117/186, 62.9%, 95% CI [55.8–69.5%]), while pus/fluid, urine, and blood each contributed a smaller proportion. Co-resistance was common, with multidrug resistance (MDR) observed in 183/186 (98.4%, 95% CI [95.4–99.4%]) and imipenem resistance in 119/186 (64.0%, 95% CI [56.9–70.5%]).

### 3.2. Resistance Phenotypes and MIC Distributions

**Figure 2:**
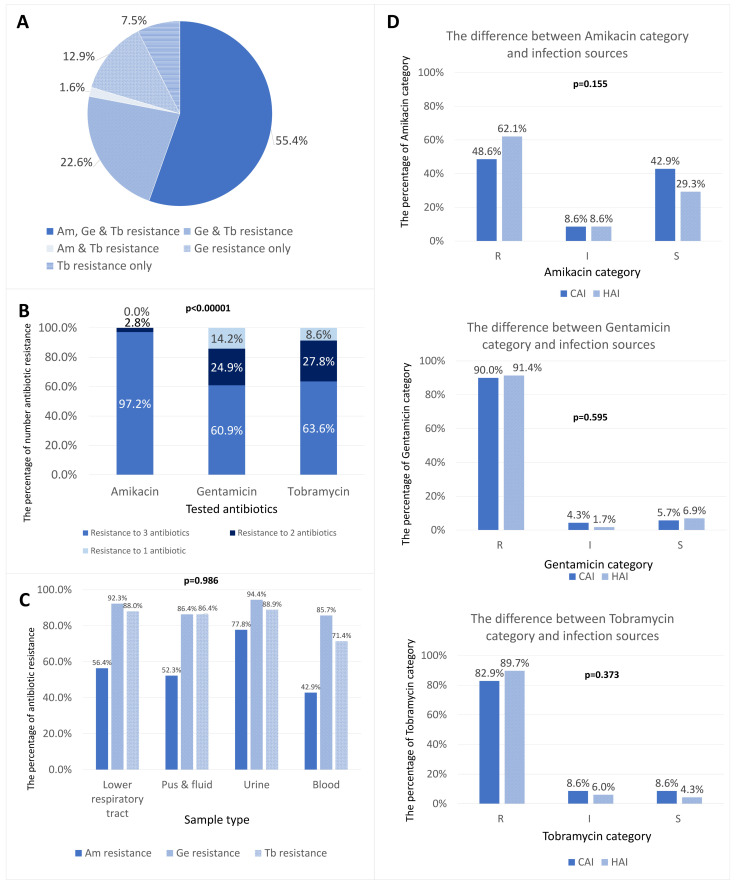
Aminoglycoside resistance phenotypes. (**A**) Combination phenotype frequencies; (**B**) Per-antibiotic resistance profiles; (**C**) Phenotypes by specimen type; (**D**) Phenotypes by infection source. Bars/violins display counts or kernel densities with the exact number of observations. Error bars are 95% CIs for proportions. S, susceptible; I, intermediate; R, resistant; CAI, community-acquired infection; HAI, hospital-acquired infection.

Among 186 isolates, aminoglycoside resistance most commonly manifested as amikacin–gentamicin–tobramycin co-resistance (103/186; 55.4% [95% CI: 48.2–62.3%]), followed by gentamicin–tobramycin co-resistance (42/186; 22.6% [95% CI: 17.2–29.1%]) while the remaining resistance patterns were less frequent (Figure 2A). The differences in resistance patterns were significant among the three agents (*p* < 0.0001) (Figure 2B), but there was no difference in antibiotic resistance distribution across sample origins (*p >* 0.05) (Figure 2C).

By infection source, susceptibility category distributions were broadly comparable between CAI and HAI for gentamicin and tobramycin, while amikacin resistance was numerically higher in HAI (*p >* 0.05) (Figure 2D).

**Figure 3:**
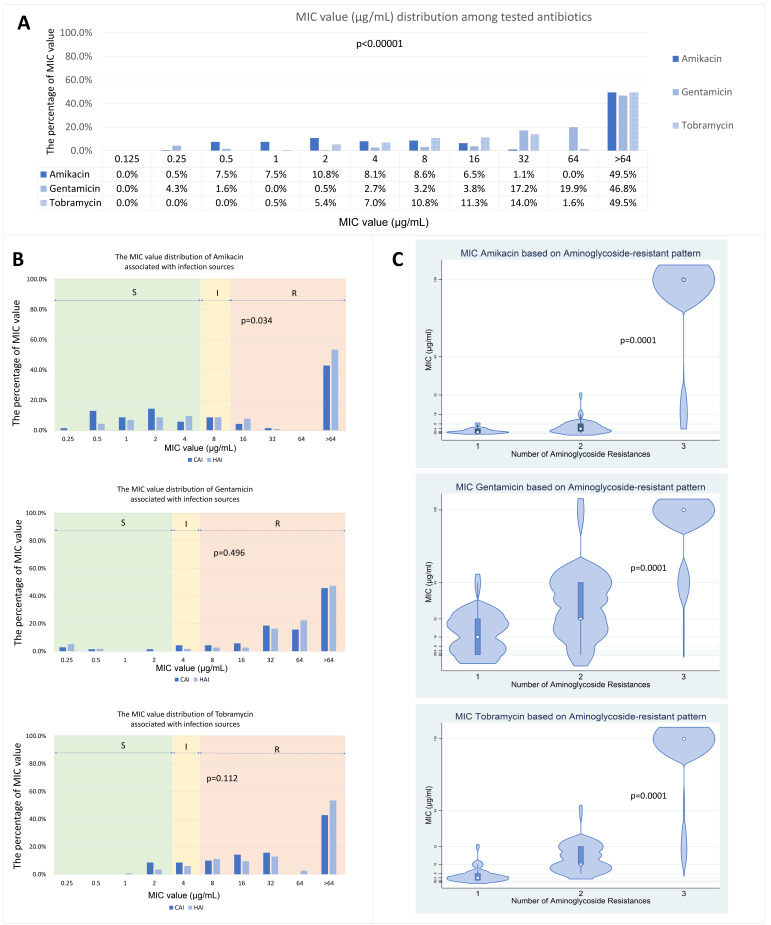
MIC distributions for amikacin, gentamicin, and tobramycin. (**A**) Distribution of MIC values for the three tested aminoglycoside antibiotics. (**B**) Differences in MIC value distributions by infection source. (**C**) Comparison of MIC value distributions by aminoglycoside resistance patterns, analyzed using the Kruskal–Wallis test. In the violin plots, the width represents the kernel density estimate, while the embedded box plot displays the interquartile range (IQR) as the central box, the median as a white circle, and whiskers extending to the data range. S, susceptible; I, intermediate; R, resistant; CAI, community-acquired infection; HAI, hospital-acquired infection. In subfigure C, the numbers 1, 2, and 3 indicate resistance to one, two, or all three aminoglycoside antibiotics tested (amikacin, gentamicin, and tobramycin), respectively.

The minimum inhibitory concentration (MIC) values for the three antibiotics tested indicated elevated resistance. Statistical analysis revealed a significant difference in MIC distributions among the antibiotics (*p* < 0.00001), with Ge exhibiting higher median MIC values compared to Am and Tb (Figure 3A). Analysis by infection source showed that HAI had significantly higher MIC values for Am than CAI (*p* = 0.034); however, no significant differences were observed for Ge or Tb (Figure 3B). Additionally, MIC values for all antibiotics were significantly associated with aminoglycoside resistance patterns. Median MIC distributions increased with resistance pattern complexity, with the highest values observed in isolates resistant to all three antibiotics (Figure 3C).

### 3.3. Aminoglycoside-Modifying Enzyme (AME) Gene Proportions

**Figure 4:**
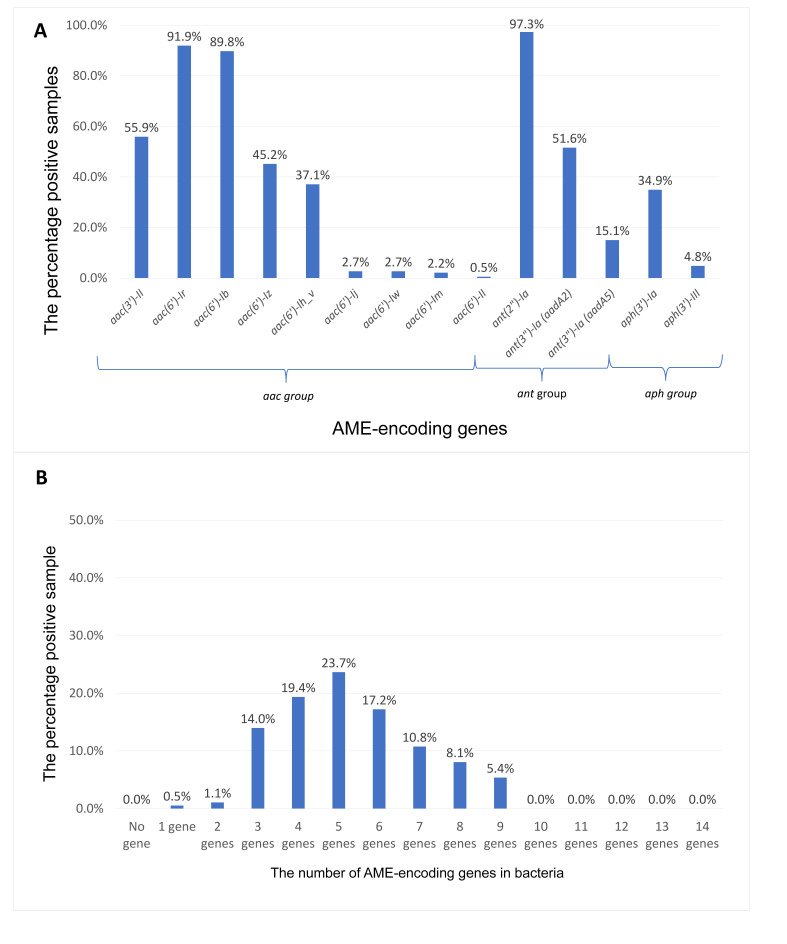
AME gene proportion and gene burden per isolate. (**A**) Proportion positive for each AME target with 95% CIs. (**B**) Distribution of the cumulative number of AME genes per isolate.

Among 186 clinical isolates, the most common genes were *ant(2″)-Ia* (181/186, 97.3% [95% CI: 94.3–98.8%]), *aac(6′)-Ir* (171/186, 91.9% [95% CI: 87.3–95.1%]), and *aac(6′)-Ib* (167/186, 89.8% [95% CI: 84.9–93.2%]) (Figure 4A). Several other determinants were detected at moderate frequencies, including the clinically relevant *aac(6′)-Ih_v* (69/186, 37.1% [95% CI: 30.6–44.0%]), whereas the remaining targets were less frequent. AME determinants were ubiquitous (0/186, 0.0% without AME genes [95% CI: 0.0–2.0%]) (Figure 4B). The within-isolate AME gene burden was high (mean 5.32 ± 1.74 [95% CI: 5.07–5.57]), with ≥5 AME genes present in 121/186 isolates (65.1% [95% CI: 58.0–71.5%]). Collectively, these data indicate widespread, multi-determinant aminoglycoside resistance potential driven by concurrent *ant*, *aac*, and *aph* genes in this cohort.

### 3.4. Associations Between AME Genes and Resistance Profiles or MICs

#### 3.4.1. Gene Proportion vs. Resistance Profiles (FDR-Adjusted)

After adjustments for multiple comparisons (Benjamini–Hochberg; false discovery rate [FDR] correction; q < 0.05), significant distinctions were observed in several gene–phenotype associations (Table 2). For amikacin resistance (Am-R vs. Am-non-R), *aac(6′)-Ib* and *aac(6′)-Ih_v* were enriched among Am-R isolates, whereas *aac(3′)-II* and *aph(3′)-Ia* were more common among Am-non-R isolates. The strongest enrichment was observed for *aac(6′)-Ih_v* (difference +34.4 percentage points, 95% CI [+15.2–+50.4]; q < 0.05).

For imipenem resistance (Imi-R vs. Imi-non-R), *aac(6′)-Ih_v* was more frequent among Imi-R isolates (difference +23.0 percentage points, 95% CI [+3.0–+40.3]; q < 0.05), while *aac(3′)-II* and *aph(3′)-Ia* were less frequent in Imi-R than Imi-non-R (Table 2). *aac(6′)-Ib* was also more common in Imi-R, but the effect estimate was smaller and should be interpreted cautiously in the context of multiple testing. Note that the non-resistant group includes isolates classified as intermediate and susceptible.

#### 3.4.2. Gene Presence vs. MIC Distributions (FDR-Adjusted)

For MIC–gene comparisons, multiple-testing correction was performed using the Benjamini–Hochberg procedure (FDR correction; q < 0.05). Median values and interquartile ranges (IQRs) in µg/mL are presented below; values denoted as “>64” are coded as 128 µg/mL for analysis. Notable MIC–gene associations were identified (Table 3). Presence of *aac(6′)-Ib* and *aac(6′)-Ih_v* was associated with higher MIC distributions across amikacin, gentamicin, and tobramycin, whereas *aac(3′)-II* and *aph(3′)-Ia* were associated with lower MIC distributions (q < 0.05). To illustrate effect magnitude, isolates carrying *aac(6′)-Ib* had substantially higher amikacin MICs than those lacking *aac(6′)-Ib* (median 128 [IQR 4–128] vs. 1 [0.5–2] µg/mL). Additional significant MIC shifts were observed for *aadA2* and *aac(6′)-Iw*, but these were less frequent determinants.

Using a diagnostic-performance framework, we evaluated whether selected AME signatures could classify key phenotypes in this cohort and reported sensitivity, specificity, positive predictive value (PPV), negative predictive value (NPV), and likelihood ratios (Supplementary Table S4). Overall, *aac(6′)-Ih_v* showed the most favorable balance of sensitivity and specificity for identifying amikacin resistance and high-level amikacin MIC categories, whereas *aac(6′)-Ib* tended to be more sensitive but less specific, consistent with its high background prevalence.

## 4. Discussion

Even though aminoglycosides are “old” antibiotics, they retain clinical value as rapidly bactericidal agents used in selected indications and frequently as combination partners against severe Gram-negative infections, with modern practice emphasizing individualized dosing and monitoring to balance efficacy and nephro-/ototoxicity risks. This relevance is amplified for *K. pneumoniae* because carbapenem-resistant *K. pneumoniae* is ranked at the top of the WHO Bacterial Priority Pathogens List 2024, reflecting its global clinical impact and constrained therapeutic options [1]. Recent Vietnamese hospital data also indicate a high prevalence of resistance among *K. pneumoniae* isolates (notably to third-generation cephalosporins and carbapenems) and demonstrate that antimicrobial resistance is associated with measurable healthcare utilization burdens, reinforcing the need to preserve any remaining effective, accessible options, including aminoglycosides when susceptibility permits [19,20]. Among aminoglycoside-resistant *Klebsiella pneumoniae*, AME genes are central determinants because they encode drug-inactivating enzymes, including acetyltransferases (AACs), nucleotidyltransferases (ANTs), and phosphotransferases (APHs), that chemically modify aminoglycosides and diminish antibacterial activity [8]. Accordingly, profiling AME genes remains highly relevant in contemporary *K. pneumoniae* epidemiology, as tertiary-hospital collections of multidrug-resistant *K. pneumoniae* continue to show substantial burdens of aminoglycoside resistance genes, supporting molecular surveillance as a practical complement to routine antimicrobial susceptibility testing [8]. In this tertiary hospital cohort in southern Vietnam, we combined standardized broth microdilution MICs with targeted profiling of 14 AME genes across 186 aminoglycoside-resistant isolates from community- and hospital-acquired infections, enabling concise genotype–phenotype assessment relevant to local surveillance and stewardship.

This study recorded that *ant(2″)-Ia* and *aac(6′)-Ir* were highly prevalent among aminoglycoside-resistant *K. pneumoniae* (Figure 4). This AME landscape aligns with global reports that identify *aac(6′)-Ib*, *ant(2″)-Ia*, and *aph(3′)-Ia* among the most prevalent determinants in *K. pneumoniae* and other Enterobacterales, yet our frequencies sit at the upper range of international estimates [8,21,22]. Differences from studies in Egypt, South Asia, and the Middle East, especially in tertiary-care settings with dense antimicrobial exposure, likely reflect heterogeneity in local antibiotic use (e.g., aminoglycoside use patterns and empiric combination therapy), infection-control infrastructure, and the underlying plasmidome/transposon repertoire [8,21,23,24,25,26,27]. Within Vietnam, genomic and epidemiologic data highlight extensive plasmid-mediated gene flow, including in hypervirulent lineages, providing a plausible conduit for widespread AME dissemination and for the coupling of AMEs with β-lactam or carbapenem resistance loci [11,26,28,29,30].

By pairing MIC testing with targeted gene detection and stratifying analyses by infection source and co-resistance backgrounds, we were able to identify AME signatures most consistently associated with elevated MIC distributions and broader resistance phenotypes.

We observed significant associations between *aac(6′)-Ib* and *aac(6′)-Ih_v* with high MICs and multidrug resistance, supporting these determinants as high-priority markers of intensified aminoglycoside resistance and broader co-resistance backgrounds in this setting (Table 2 and Table 3). The strong, directionally consistent link suggests a broad-spectrum impact, plausibly reflecting high gene expression and/or co-carriage with other resistance determinants on mobile elements. At the same time, multiple recent studies underscore that very high aminoglycoside MICs are often driven primarily by acquired 16S rRNA methyltransferases (e.g., *armA,rmtB/rmtC/rmtD*), which are frequently co-located with multiple AMEs on conjugative plasmids, warranting targeted screening for these determinants in follow-on work [31,32,33]. These associations have practical implications for both clinical decision-making and Low- and Middle-Income Countries (AMR) surveillance. A focused set of “high-risk” AME signatures may serve as a rapid adjunct to phenotypic testing, supporting early risk stratification and prioritization of confirmatory MIC testing when culture-based results are delayed. In this study, the observed diagnostic performance of *aac(6′)-Ih_v* (and, to a lesser extent, *aac(6′)-Ib)* suggests these targets could be deployed as an early warning signal for this purpose (Supplementary Table S4). Moreover, because *aac(6′)-Ib* variants may co-travel with resistance to other drug classes (notably *aac(6′)-Ib-cr* and quinolone non-susceptibility), monitoring these AME constellations can function as an early-warning proxy for broader MDR risk and help prioritize infection-prevention responses in tertiary-care settings [21,34].

In contrast, *aac(3′)-II*, *aadA2*, *aph(3′)-Ia*, and *aac(6′)-Iw* tended to accompany lower MICs (Table 3). This observation is concordant with genotype-phenotype analyses showing that several AME classes exert only modest effects on aminoglycoside susceptibility unless combined with stronger determinants, reinforcing the value of interpreting AMEs as combinatorial predictors rather than as uniformly high-impact markers [32,34].

We observed higher amikacin MICs in HAI compared with CAI (Figure 3), although AME gene frequencies did not differ significantly after multiple-testing adjustment (Table 2). The finding of higher amikacin MICs in HAI is also consistent with the broader pattern that hospital cohorts frequently contain dense resistance gene backgrounds and high aminoglycoside non-susceptibility, even when single-gene frequency differences are not always apparent after correction for multiple testing [8,35]. The absence of robust CAI-HAI differences in gene prevalence (despite higher amikacin MICs in HAI) supports the hypothesis that similar AME pools circulate across clinical compartments. Still, hospital-selective pressures (e.g., broader empiric regimens, prolonged exposure) amplify the intensity of phenotypic resistance.

HAI settings impose intense selective pressures that can shift aminoglycoside MIC distributions upward because critically ill patients are frequently exposed to empiric broad-spectrum therapy, combination regimens, prolonged treatment courses, and high-intensity care environments that facilitate repeated antibiotic “cycling” and within-unit amplification of resistant subpopulations [36]. In Vietnamese critical-care units, empirical antibiotics were initiated within 24 h in 63.6% of admissions; 31.5% of patients received antibiotics despite no infection diagnosis; Watch/Reserve agents comprised 87.3% of early prescriptions; and cultures were obtained in only 12.9% of treated patients [37]. A cluster-randomized trial in 16 district hospitals also found that inappropriate prescribing exceeded 60% at baseline and that a multifaceted stewardship package reduced inappropriate prescribing by 6.3% (difference-in-differences vs. control), providing directly actionable evidence that hospital-driven selection pressure is modifiable in Low- and Middle-Income Countries (LMIC) contexts [38]. This practice leads to an exposure profile that plausibly intensifies hospital selection for higher-MIC phenotypes, even when the underlying resistance-gene pool is broadly shared. Complementing these clinical observations, a prospective genomic epidemiology study in Hanoi hospitals has documented extensive endemicity and spread of MDR *Klebsiella pneumoniae* with diverse plasmid-mediated resistance determinants, consistent with similar AME pools circulating across compartments while hospital exposures and co-resistance backgrounds preferentially enrich high-level phenotypes [39]. From a practice and research standpoint, these data argue for pairing phenotypic surveillance with standardized antimicrobial-use measurement and AWaRe-informed targets (including the ≥60% Access consumption target) to operationalize stewardship, quantify selection pressure, and design CAI-HAI studies that explicitly model prior exposure, length of stay, and transmission covariates [40,41].

Molecular detection of AME genes can accelerate recognition of aminoglycoside resistance. Still, genotype-to-phenotype inference remains probabilistic because AME families are drug- and allele-specific, and the surrounding resistance background modulates their phenotypic effect [42]. Contemporary phenotyping of diverse *aac(6′)* alleles shows that closely related variants can shift substrate profiles across aminoglycosides (e.g., amikacin vs. gentamicin vs. tobramycin), meaning that coarse “gene-family present” calls can over- or under-estimate activity for a specific aminoglycoside. In isogenic backgrounds, acquisition of individual AMEs (e.g., AAC/ANT/APH enzymes) can drive significant MIC shifts, yet these effects can coexist with (and be partly masked or amplified by) non-enzymatic mechanisms such as aminoglycoside efflux, underscoring that phenotype is frequently multi-mechanistic rather than a single-marker trait. Clinical isolate datasets similarly show that AME carriage correlates strongly, but not perfectly, with aminoglycoside resistance because additional mechanisms (efflux regulation, biofilm-associated tolerance, permeability, and occasional 16S rRNA methylation) contribute to the observed MIC distribution [43]. In carbapenem-resistant *Klebsiella pneumoniae*, multigene AME signatures can predict aminoglycoside MICs with high but incomplete agreement, leaving clinically relevant residual error for isolate-level decision-making (including for amikacin and tobramycin) [42]. Importantly, aminoglycoside heteroresistance can create low-frequency subpopulations with higher amikacin MICs that routine workflows may miss, and copy-number–linked overexpression of AME determinants (including *aac(6′)* variants) has been shown to generate transient aminoglycoside heteroresistance that rapidly enriches under antibiotic exposure [44,45]. Moreover, when 16S rRNA methyltransferases (e.g., *armA/rmt* family) are present, they are strong indicators of broad/high-level aminoglycoside non-susceptibility and often co-travel with other hospital-adapted resistance determinants, narrowing the utility of aminoglycosides, even for newer agents [42,46]. Therefore, AME-focused molecular results should be interpreted as complementary to phenotypic antimicrobial susceptibility tests (ideally MIC-based), with explicit aminoglycoside-specific interpretive rules, awareness of allele-level differences, and ongoing local validation of genotype–phenotype performance in prevalent lineages before using gene detection alone to guide therapy or stewardship metrics [42,47].

The single-center, cross-sectional design limits generalizability and precludes inference on transmission dynamics. Our qPCR panel, while broad, could not capture AME variants outside the targeted primer set or alternative mechanisms, resulting in an underestimation of resistance diversity. Lack of whole-genome sequencing (WGS) constrained assessment of plasmid backbones, integrons, and co-localization with virulence genes. Clinical outcome data (e.g., mortality, length of stay) were unavailable, preventing evaluation of the patient-level impact of specific AME profiles. These factors temper external validity and argue for cautious extrapolation beyond similar Vietnamese tertiary-care environments.

Surveillance and infection control measures are central to preventing antimicrobial resistance, particularly given the high prevalence of AME carriage among Vietnamese *K. pneumoniae*. This underscores the need for nationwide molecular surveillance integrated with routine microbiology practices. Regular AME screening with tiered qPCR panels should be performed on clinically significant isolates from healthcare-associated infections and high-risk wards to promptly detect evolving resistance risks. Complementing this, whole-genome sequencing of sentinel isolates can reveal the genetic contexts of AME genes, such as plasmids, integrons, and transposons, and shed light on co-selection with ESBL/carbapenemase loci, thereby providing actionable data for outbreak investigation and containment. Integrating surveillance findings into antimicrobial stewardship programmes is equally crucial, necessitating the inclusion of AME profiles and local MIC distributions in empiric therapy recommendations and antibiograms; notably, aminoglycosides should be used cautiously in environments dominated by *aac(6′)-Ib* and *aac(6′)-Ih_v* unless evidence-based regimens demonstrate efficacy.

Research priorities focus on establishing multicentre Vietnamese cohorts to monitor national trends, conducting functional studies on *aac(6′)-Ib* and *aac(6′)-Ih_v* expression and associated fitness costs, and mapping the resistome and virulome to clarify links with hypervirulence and clinical outcomes. Judicious use of empiric aminoglycoside therapy, limited to short courses and informed by molecular and phenotypic data, with early de-escalation and optimised dosing via therapeutic drug monitoring and combination strategies, is advised in high-burden wards. Rapid integration of molecular results into stewardship workflows can expedite effective therapy initiation while minimising unnecessary exposure.

## 5. Conclusions

In this Vietnamese tertiary-care setting, *ant(2″)-Ia* and *aac(6′)-Ir* were highly prevalent in aminoglycoside-resistant *Klebsiella pneumoniae*, and *aac(6′)-Ib* and *aac(6′)-Ih_v* were consistently associated with higher MICs and multidrug resistance. AME gene frequencies did not differ significantly between CAI and HAI, suggesting that hospital-level selective pressures may intensify phenotypic resistance without detectably altering gene prevalence.

Multicentre studies across Vietnam with WGS-enabled surveillance are warranted to establish national baselines and delineate mobile genetic elements. The high AME burden and gene-specific links to high-level resistance provide actionable intelligence to support risk-stratified surveillance and stewardship decisions, particularly in tertiary-care settings where co-resistance is common.

## Figures and Tables

**Table 1:** Study population characteristics.

Aminoglycoside-Resistant *K. pneumoniae* Isolates Characteristics (N = 186)			N	% [95% CI]
Clinical pattern	Infection sources	CAI	70	37.6% [31.0–44.8%]
		HAI	116	62.4% [55.2–69.0%]
	Specimen types	Lower respiratory tract	117	62.9% [55.8–69.5%]
		Pus and fluid	43	23.1% [17.6–29.7%]
		Urine	19	10.2% [6.6–15.4%]
		Blood	7	3.8% [1.8–7.6%]
Antibiotic resistance pattern	Multidrug resistance		183	98.4% [95.4–99.4%]
	ESBL production		55	29.6% [23.5–36.5%]
	Imipenem resistance		119	64.0% [56.9–70.5%]

CAI, Community-acquired infection; HAI, Hospital-acquired infection.

**Table 2:** Prevalence of AME Genes stratified by infection source and resistance profiles.

	The AMEs Encoding Genes in Study Population (n, %)														
	*aac* Group									*ant* Group			*aph* Group		Total n (%)
	*aac(3′)-II*	*aac(6′)-Ib*	*aac(6′)-Ir*	*aac(6′)-Iz*	*aac(6′)-Ih_v*	*aac(6′)-Ij*	*aac(6′)-Iw*	*aac(6′)-Im*	*aac(6′)-II*	*ant(2″)-Ia*	*ant(3″)-I (aadA2)*	*ant(3″)-I (aadA5)*	*aph(3′)-Ia*	*aph(3′)-III*	
**Infection sources**															
CAI	40 (57.1%)	62 (88.6%)	64 (91.4%)	31 (44.3%)	20 (28.6%)	2 (2.9%)	3 (4.3%)	2 (2.9%)	0(0.0%)	68 (97.1%)	38 (54.3%)	15 (21.4%)	31 (44.3%)	3 (4.3%)	70 (100%)
HAI	64 (55.2%)	105 (90.5%)	107 (92.2%)	53 (45.7%)	49 (42.2%)	3 (2.6%)	2 (1.7%)	2 (1.7%)	1 (0.9%)	113 (97.4%)	58 (50.0%)	13 (11.2%)	34 (29.3%)	6 (5.2%)	116 (100%)
*p*	0.793	0.671	0.844	0.852	0.062	1.000	0.366	0.632	1.000	1.000	0.571	0.059	0.038	1.000	
**Resistance profiles**															
Am resistant	41 (38.7%)	102 (96.2%)	97 (91.5%)	46 (43.4%)	55 (51.9%)	2 (1.9%)	0(0.0%)	1 (0.94%)	1 (0.94%)	104 (98.1%)	46 (43.4%)	13 (12.3%)	25 (23.6%)	5 (4.72%)	106 (100%)
Am non-resistant	63 (78.8%)	65 (81.3%)	74 (92.5%)	38 (47.5%)	14 (17.5%)	3 (3.8%)	5 (6.3%)	3 (3.8%)	0(0.0%)	77 (96.3%)	50 (62.5%)	15 (18.8%)	40 (50.0%)	4 (5.0%)	80 (100%)
*p*	**<0.0001**	**0.001**	0.806	0.578	**<0.0001**	0.653	0.014	0.316	1.000	0.653	1.000	0.221	**<0.0001**	1.000	
Ge resistant	98 (58.0%)	150 (88.8%)	155 (91.7%)	76 (45.0%)	63 (37.3%)	5 (3.0%)	5 (3.0%)	4 (2.4%)	1 (0.6%)	165 (97.6%)	86 (50.9%)	26 (15.4%)	61 (36.1%)	9 (5.3%)	169 (100%)
Ge non-resistant	6 (35.3%)	17 (100%)	16 (94.1%)	8 (47.1%)	6 (35.3%)	0(0.0%)	0(0.0%)	0(0.0%)	0(0.0%)	16 (94.1%)	10 (58.8%)	2 (11.8%)	4 (23.5%)	0(0.0%)	17 (100%)
*p*	0.072	0.225	1.000	0.869	0.872	1.000	1.000	1.000	1.000	0.384	0.533	1.000	0.300	1.000	
Tb resistant	81 (50.0%)	153 (94.4%)	149 (92.0%)	76 (46.9%)	67 (41.4%)	3 (1.9%)	4 (2.5%)	4 (2.5%)	1 (0.6%)	157 (96.9%)	80 (49.4%)	23 (14.2%)	49 (30.3%)	9 (5.6%)	162 (100%)
Tb non-resistant	23 (95.8%)	14 (58.3%)	22 (91.7%)	8 (33.3%)	2 (8.3%)	2 (8.3%)	1 (4.2%)	0(0.0%)	0(0.0%)	24 (100%)	16 (66.7%)	5 (20.8%)	16 (66.7%)	0(0.0%)	24 (100%)
*p*	**<0.0001**	**<0.0001**	1.0000	0.212	**0.002**	0.125	0.503	1.000	1.000	1.000	0.114	0.370	**0.001**	0.607	
**The number of resistant antibiotics per strain**															
Resistance to 3 antibiotics	39 (37.9%)	99 (96.1%)	94 (91.3%)	44 (42.7%)	52 (50.5%)	2 (1.9%)	0(0.0%)	1 (1.0%)	1 1.0%)	101 (98.1%)	43 (41.8%)	13 (12.6%)	25 (24.3%)	5 (4.9%)	103 (100%)
Resistance to 2 antibiotics	38 (84.4%)	40 (88.9%)	42 (93.3%)	26 (57.8%)	12 (26.7%)	1 (2.2%)	4 (8.9%)	3 (6.7%)	0(0.0%)	43 (95.6%)	30 (66.7%)	8 (17.8%)	20 (44.4%)	4 (8.9%)	45 (100%)
Resistance to 1 antibiotic	27 (71.1%)	28 (73.7%)	35 (92.1%)	14 (36.8%)	5 (13.2%)	2 (5.3%)	1 (2.6%)	0(0.0%)	0(0.0%)	37 (97.4%)	23 (60.5%)	7 (18.4%)	20 (52.6%)	0(0.0%)	38 (100%)
*p*	**<0.0001**	**0.001**	1.000	0.122	**<0.0001**	0.407	**0.006**	0.086	-	0.715	**0.010**	0.584	**0.002**	0.190	
**ESBL-producing ability**															
ESBL producing	39 (70.9%)	51 (92.7%)	52 (94.6%)	29 (52.7%)	18 (32.7%)	4 (7.3%)	5 (9.1%)	2 (3.6%)	0(0.0%)	53 (96.4%)	29 (52.7%)	13 (23.6%)	27 (49.1%)	3 (5.5%)	55 (100%)
Non-ESBL producing	65 (49.6%)	116 (88.6%)	119 (90.8%)	55 (42.0%)	51 (38.9%)	1 (0.8%)	0(0.0%)	2 (1.5%)	1 (0.8%)	128 (97.7%)	67 (51.2%)	15 (11.5%)	38 (29.0%)	6 (4.6%)	131 (100%)
*p*	**0.008**	0.391	0.0397	0.199	0.424	0.027	**0.002**	0.583	1.000	0.633	0.844	0.034	0.012	0.725	
**Imipenem-resistant ability**															
Imipenem resistant	54 (45.4%)	112 (94.1%)	107 (89.9%)	54 (45.4%)	54 (45.4%)	1 (0.8%)	1 (0.8%)	2 (1.7%)	1 (0.8%)	116 (97.5%)	52 (43.7%)	15 (12.6%)	31 (26.1%)	7 (5.9%)	119 (100%)
Imipenem non-resistant	50 (74.6%)	55 (82.1%)	64 (95.5%)	30 (44.8%)	15 (22.4%)	4 (6.0%)	4 (6.0%)	2 (3.0%)	0(0.0%)	65 (97.0%)	44 (65.7%)	13 (19.4%)	34 (50.8%)	2 (3.0%)	67 (100%)
*p*	**<0.0001**	**0.011**	0.263	0.937	**0.002**	0.057	0.057	0.62	1.000	1.000	**0.004**	0.213	**0.001**	0.492	

Chi-square and two side-Fisher’s exact tests with Benjamini–Hochberg procedure, adjusted *p* ≤ 0.011 were considered statistically significant. Non-resistant category includes intermediate and susceptible strains.

**Table 3:** Distribution of Aminoglycoside MICs stratified by presence or absence of AME genes.

AME Gene N = 186			Amikacin			Gentamicin			Tobramycin		
			Median (IQR) (µg/mL)	MIC > 64 (µg/mL) (N, %)	*p*	Median (IQR) (µg/mL)	MIC > 64 (µg/mL) (N, %)	*p*	Median (IQR) (µg/mL)	MIC > 64 (µg/mL) (N, %)	*p*
***aac(3′)-II***	Presence	N = 104	8 (2–128)	28 (26.9%)	**<0.0001**	64 (32–128)	28 (26.9%)	**<0.0001**	32 (8–128)	28 (26.9%)	**<0.0001**
	Absence	N = 82	128 (128–128)	64 (78.0%)		128 (64–128)	59 (72.0%)		128 (128–128)	64 (78.0%)	
***aac(6′)-Ib***	Presence	N = 167	128 (4–128)	88 (52.7%)	**<0.0001**	64 (32–128)	82 (49.1%)	**0.0076**	128 (16–128)	88 (52.7%)	**0.0001**
	Absence	N = 19	1 (0.5–2)	4 (21.1%)		32 (16–128)	5 (26.3%)		4 (2–32)	4 (21.1%)	
***aac(6′)-Ij***	Presence	N = 5	8 (1–16)	1 (20.0%)	0.218	64 (32–64)	1 (20.0%)	0.534	32 (4–32)	1 (20.0%)	0.19
	Absence	N= 181	128 (2–128)	91 (50.3%)		64 (32–128)	86 (47.5%)		128 (16–128)	91 (50.3%)	
***aac(6′)-Im***	Presence	N = 4	4 (3–66)	1 (25.0%)	0.422	48 (32–96)	1 (25.0%)	0.569	32 (20–80)	1 (25.0%)	0.673
	Absence	N = 182	80 (2–128)	91 (50.0%)		64 (32–128)	86 (47.3%)		96 (16–128)	91 (50.0%)	
***aac(6′)-Ih_v***	Presence	N = 69	128 (16–128)	50 (72.5%)	**<0.0001**	128 (64–128)	44 (63.8%)	**0.0003**	128 (32–128)	50 (72.5%)	**<0.0001**
	Absence	N = 117	8 (2–128)	42 (35.9%)		64 (32–128)	43 (36.8%)		32 (8–128)	42 (35.9%)	
***aac(6′)-Iw***	Presence	N = 5	2 (0.5–2)	0 (0.0%)	**0.0071**	32 (32–32)	0 (0.0%)	0.019	8 (8–8)	0 (0.0%)	**0.012**
	Absence	N = 181	128 (4–128)	92 (50.8%)		64 (32–128)	87 (48.1%)		128 (16–128)	92 (50.8%)	
***aac(6′)-Iz***	Presence	N = 84	16 (2–128)	39 (46.4%)	0.634	64 (32–128)	37 (44.0%)	0.781	32 (16–128)	39 (46.4%)	0.803
	Absence	N = 102	128 (2–128)	53 (52.0%)		64 (32–128)	50 (49.0%)		128 (16–128)	53 (52.0%)	
***aac(6′)-Ir***	Presence	N = 171	16 (2–128)	83 (48.5%)	0.421	64 (32–128)	77 (45.0%)	0.341	64 (16–128)	83 (48.5%)	0.535
	Absence	N= 15	128 (4–128)	9 (60.0%)		128 (32–128)	10 (66.7%)		128 (16–128)	9 (60.0%)	
***aac(6′)-II***	Presence	N = 1	128	1 (100.0%)	0.350	128	1 (100.0%)	0.327	128	1 (100.0%)	0.349
	Absence	N = 185	32 (2–128)	91 (49.2%)		64 (32–128)	86 (46.5%)		64 (16–128)	91 (49.2%)	
***ant(2″)-Ia***	Presence	N = 181	32 (2–128)	90 (49.7%)	1.000	64 (32–128)	85 (47.0%)	0.537	64 (16–128)	90 (49.7%)	0.964
	Absence	N = 5	8 (4–128)	2 (40.0%)		64 (16–128)	2 (40.0%)		32 (32–128)	2 (40.0%)	
***aadA2***	Presence	N = 96	8 (2–128)	35 (36.5%)	**0.0004**	64 (32–128)	34 (35.4%)	**0.0019**	32 (8–128)	35 (36.5%)	**0.001**
	Absence	N = 90	128 (4–128)	57 (63.3%)		128 (64–128)	53 (58.9%)		128 (16–128)	57 (63.3%)	
***aadA5***	Presence	N = 28	8 (2–128)	13 (46.4%)	0.356	64 (32–128)	13 (46.4%)	0.9645	32 (12–128)	13 (46.4%)	0.556
	Absence	N = 158	80 (2–128)	79 (50.0%)		64 (32–128)	74 (46.8%)		96 (16–128)	79 (50.0%)	
***aph (3′)-Ia***	Presence	N = 65	4 (1–128)	22 (33.8%)	**<0.0001**	64 (32–128)	21 (32.3%)	**0.0034**	32 (8–128)	22 (33.8%)	**0.0002**
	Absence	N = 121	128 (8–128)	70 (57.9%)		128 (64–128)	66 (54.5%)		128 (16–128)	70 (57.9%)	
***aph (3′)-III***	Presence	N = 9	128 (8–128)	5 (55.6%)	0.714	128 (64–128)	5 (55.6%)	0.355	128 (32–128)	5 (55.6%)	0.385
	Absence	N = 177	32 (2–128)	87 (49.2%)		64 (32–128)	82 (46.3%)		64 (16–128)	87 (49.2%)	

MIC normality assessed via Shapiro–Wilk test (*p* < 0.05 indicates significance). IQR: Interquartile range; The value of “>64” was converted to 128 µg/mL for calculating median and IQR. The Mann–Whitney U test with Benjamini–Hochberg procedure, adjusted *p* ≤ 0.019 were considered statistically significant.

## Data Availability

All original contributions reported in this study are included in the article. Additional inquiries should be addressed to the corresponding author.

## Supplementary Materials

The following supporting information can be downloaded at: https://www.mdpi.com/article/10.3390/microorganisms14020463/s1, Table S1: Complete primer sequences and cycling conditions for qPCR; Table S2: Manufacturers and catalogue numbers for key reagents/consumables and instruments for bacterial culture, DNA extraction and qPCR; Table S3: Manufacturers and catalogue numbers for key reagents/consumables for MIC determination; Table S4: Diagnostic performance of selected AME genes for predicting resistance phenotypes.

## Abbreviations

AMEs, Aminoglycoside Modifying Enzymes; ATCC, American Type Culture Collection; AST, Antibiotic susceptibility testing; CLSI, Clinical and Laboratory Standards Institute guidelines; Am, Amikacin; Ge, Gentamicin; Tb, Tobramycin; PCR, polymerase-chain reaction; NTC, non-template control; MIC, Minimum Inhibitory Concentration.
