# Supplementary material for: Genotype–Phenotype Links Between Aminoglycoside-Modifying Enzymes and Aminoglycoside MICs in Aminoglycoside-Resistant Klebsiella pneumoniae in a Southern Vietnam Tertiary Hospital
Source: Microorganisms. 2026 Feb 13;14(2):463. doi: 10.3390/microorganisms14020463 (PMC12942977; doi:10.3390/microorganisms14020463)
Supplement: Supplementary file 1 [file microorganisms-14-00463-s001.zip › Supplementary Table S2 _ Manufacturers and catalogue numbers for key reagents and instruments for DNA extraction and ZKIR qPCR .pdf]

Supplementary Table S2. Manufacturers and catalogue numbers for key reagents/consumables and instruments for bacterial culture, DNA extraction and qPCR

No.	Reagents/consumables and instruments	Catalogue number	Corresponding company name	Purpose
1	BHI Broth	M210-500G	Himedia (India)	Baterial culture
2	TopPURE® GENOMIC DNA EXTRACTION KIT	HE-001	ABT (VietNam)	DNA extraction
3	SensiFAST™ SYBR® Lo-ROX Kit	BIO-94005	Bioline (UK)	qPCR
4	MicroAmp™ Optical 384-Well Reaction Plate with Barcode	262160	Thermo Fisher Scientific (US)	qPCR
5	QuantStudio 12K Flex Real-Time PCR System	-	Thermo Fisher Scientific (US)	qPCR
6	QuantStudio™ 5 Real-Time PCR System	-	Thermo Fisher Scientific (US)	qPCR
