# Supplementary material for: Genotype–Phenotype Links Between Aminoglycoside-Modifying Enzymes and Aminoglycoside MICs in Aminoglycoside-Resistant Klebsiella pneumoniae in a Southern Vietnam Tertiary Hospital
Source: Microorganisms. 2026 Feb 13;14(2):463. doi: 10.3390/microorganisms14020463 (PMC12942977; doi:10.3390/microorganisms14020463)
Supplement: Supplementary file 1 [file microorganisms-14-00463-s001.zip › Supplementary Table S3_Manufacturers and catalogue numbers for key reagents and instruments for MIC determination.pdf]

Supplementary Table S3. Manufacturers and catalogue numbers for key reagents/consumables for MIC determination

No.	Reagents/consumables	Catalogue number	Corresponding company name
1	BBL™ Mueller Hinton II Broth (Cation-adjusted)	212322	Becton Dickinson (US)
2	Resazurin sodium salt	R7017-1G	Sigma-Aldrich (US)
3	Amikacin disulfate salt	A1774-250MG	
4	Gentamicin sulfate salt	G3632-1G	
5	Tobramycin sulfate salt	T1783-100MG	
6	Corning® 96-well Clear Flat Bottom Polystyrene TC-treated Microplates, Individually Wrapped, with Lid, Sterile	3596	Corning (US)

Antibiotics were dissolved in ddH_2O, and the final concentrations tested ranged from 0.125 to 64 μg/mL. Resazurin was dissolved in PBS (pH=7.4) to a final concentration of 0.15 mg/mL.
