# Supplementary material for: Genotype–Phenotype Links Between Aminoglycoside-Modifying Enzymes and Aminoglycoside MICs in Aminoglycoside-Resistant Klebsiella pneumoniae in a Southern Vietnam Tertiary Hospital
Source: Microorganisms. 2026 Feb 13;14(2):463. doi: 10.3390/microorganisms14020463 (PMC12942977; doi:10.3390/microorganisms14020463)
Supplement: Supplementary file 1 [file microorganisms-14-00463-s001.zip › Supplementary_Table S1_Complete primer sequences and cycling conditions for qPCR.pdf]

Supplementary Table S1. Complete primer sequences and cycling conditions for qPCR

For ZKIR qPCR [1], each total 10 µl reaction contained 5 µl of SensiFAST™ SYBR Lo-ROX Kit (Bioline, UK), 0.5 µl of each primer (final concentration, 500 nM), 2 µl of ddH_2O, and 2 µl of DNA (20 ng total). Amplification was performed on a QuantStudio 5 Real-Time PCR System (Thermo Fisher Scientific, US) using the following protocol: 95°C for 3 minutes, followed by 40 cycles of 95°C for 10 seconds and 60°C for 1 minute, and ending with a melting curve analysis (1.6°C/s decrement).

For qPCR detection of AME genes [2], each total 5 µL reaction contained 2.5 µL of SensiFAST™ SYBR Lo-ROX Kit (Bioline, UK), 0.25 µL of each primer (final concentration, 250 nM), and 2 µL of DNA template (20 ng). Amplification was performed on a QuantStudio 12K Flex Real-Time PCR System (Thermo Fisher Scientific, US) using the following protocol: 95°C for 10 minutes, followed by 40 cycles of 95°C for 15 seconds and 65°C for 15 seconds, and ending with a melting curve analysis (1.6°C/s decrement).

Primer number in reference study	Gene target	Forward sequence (5'-3')	Reverse sequence (5'-3')
-	*ZKIR* [1]	CTAAAACCGCCATGTCCGATTTAA	TTCCGAAAATGAGACACTTCAGA
3	*aacC2 (or aac(3')-II)* [2]	ACGGCATTCTCGATTGCTTT	CCGAGCTTCACGTAAGCATTT
14	*aphA3 (or (aph(3')-III)* [2]	AAAAGCCCGAAGAGGAACTTG	CATCTTTCACAAAGATGTTGCTGTCT
95	*aac(6')-Ib* [2]	CGTCGCCGAGCAACTTG	CGGTACCTTGCCTCTCAAACC
97	*aadA2* [2]	ACGGCTCCGCAGTGGAT	GGCCACAGTAACCAACAAATCA
98	*aadA5* [2]	ATCACGATCTTGCGATTTTGCT	CTGCGGATGGGCCTAGAAG
170	*aphA1 (or aph(3')-Ia)* [2]	TGAACAAGTCTGGAAAGAAATGCA	CCTATTAATTTCCCCTCGTCAAAAA
414	*aac(6)-iic* [2]	CAGTCTTTGGCTAATCCATCACAG	AACGAACCCGGCCTTCTC
415	*aac(6)-ij* [2]	ATGCCTGTATCTGAATCCCTGATG	GGCAATCGCTTGTTGAGTATCTG
417	*aac(6)-im* [2]	CGTGAGCATTATACAGAGCAATGG	CCATTTCCGTTCGTAGATATTGGC
418	*aac(6)-ir* [2]	GCTATAACGATCAGCAGCAAGC	CGCGATGCATGGCATGAC
420	*aac(6)-ih_v* [2]	TTGGCTTATACCGACACCCA	CCCGTTGCGATACCTGAAC
421	*aac(6)-iw* [2]	TGCGTCAGTTACTTACACGAAC	CCTGATGCATTGCATGACTGA

422	*aac(6)-iz* [2]	TGCGCCATGACTACGTGAAC	GACTGTCCGAAGCCAGTTCG
429	*aadB (or ant(2')-Ia)* [2]	CCTGCTTGGTGGGCAGAC	CGGCACGCAAGACCTCAA

Reference

1. Barbier E, Rodrigues C, Depret G, Passet V, Gal L, Piveteau P, Brisse S. The ZKIR Assay, a Real-Time PCR Method for the Detection of *Klebsiella pneumoniae* and Closely Related Species in Environmental Samples. *Applied and Environmental Microbiology*. 2020;86(7).

2. Stedtfeld RD, Guo X, Stedtfeld TM, Sheng H, Williams MR, Hauschild K, Gunturu S, Tift L, Wang F, Howe A, Chai B, Yin D, Cole JR, Tiedje JM, Hashsham SA. Primer set 2.0 for highly parallel qPCR array targeting antibiotic resistance genes and mobile genetic elements. *FEMS Microbiology Ecology*. 2018;94(9).
