# Supplementary material for: Genotype–Phenotype Links Between Aminoglycoside-Modifying Enzymes and Aminoglycoside MICs in Aminoglycoside-Resistant Klebsiella pneumoniae in a Southern Vietnam Tertiary Hospital
Source: Microorganisms. 2026 Feb 13;14(2):463. doi: 10.3390/microorganisms14020463 (PMC12942977; doi:10.3390/microorganisms14020463)
Supplement: Supplementary file 1 [file microorganisms-14-00463-s001.zip › Supplementary_Table_S4_Diagnostic_Performance.pdf]

Supplementary Table S4. Diagnostic performance of selected AME genes for predicting resistance phenotypes.

Test positive = AME gene detected by qPCR. Outcome positive = phenotypic resistance (R) by CLSI breakpoints; non-R includes intermediate (I) and susceptible (S). Wilson 95% confidence intervals are shown in square brackets.

Marker	Outcome	TP	FN	FP	TN	Sensitivity (95% CI)	Specificity (95% CI)	PPV (95% CI)	NPV (95% CI)	LR+	LR-	Accuracy
aac(6')-Ib	Amikacin resistance (R vs non-R)	102	4	65	15	96.2% [90.7%–98.5%]	18.8% [11.7%–28.7%]	61.1% [53.5%–68.1%]	78.9% [56.7%–91.5%]	1.18	0.20	62.9%
aac(6')-Ih_v	Amikacin resistance (R vs non-R)	55	51	14	66	51.9% [42.5%–61.2%]	82.5% [72.7%–89.3%]	79.7% [68.8%–87.5%]	56.4% [47.4%–65.1%]	2.96	0.58	65.1%
aac(6')-Ib	Imipenem resistance (R vs non-R)	112	7	55	12	94.1% [88.4%–97.1%]	17.9% [10.6%–28.7%]	67.1% [59.6%–73.7%]	63.2% [41.0%–80.9%]	1.15	0.33	66.7%
aac(6')-Ih_v	Imipenem resistance (R vs non-R)	54	65	15	52	45.4% [36.7%–54.3%]	77.6% [66.3%–85.9%]	78.3% [67.2%–86.4%]	44.4% [35.8%–53.5%]	2.03	0.70	57.0%

Abbreviations: TP, true positives; FN, false negatives; FP, false positives; TN, true negatives; PPV, positive predictive value; NPV, negative predictive value; LR+, positive likelihood ratio; LR-, negative likelihood ratio.
